# 3D Strain helps relating LV function to LV and structure in athletes

**DOI:** 10.1186/1476-7120-12-33

**Published:** 2014-08-12

**Authors:** Laura Stefani, Alessio De Luca, Loira Toncelli, Gianni Pedrizzetti, Giorgio Galanti

**Affiliations:** 1 Sport Medicine Center, Department of Experimental and Clinical Medicine, University of Florence, Florence, Italy; 2 Department of Engineering and Architecture, University of Trieste, Trieste, Italy

**Keywords:** Three-dimensional strain, Cardiac mechanics, Physical exercise

## Abstract

**Introduction:**

The evaluation of cardiac contraction could benefit from a connection with the underlying helical structure of cardiac fibers in athletes either completely healthy or with minor common cardiopathies like Bicuspid Aortic Valve (BAV). This study aims to exploit the potential role of 3D strain to improve the physiological understanding of LV function and modification due to physical activity as a comparative model.

**Methods:**

Three age-matched groups of young (age 20.3 ± 5.4) individuals are prospectively enrolled: 15 normal healthy subjects, 15 healthy athletes, and 20 athletes with bicuspid aortic valve (BAV). All subjects underwent echocardiographic examination and both 2D and 3D strain analysis.

**Results:**

All echo parameters were within the normal range in the three groups. Global values of end-systolic longitudinal and circumferential strain, assesses by either 2D or 3D analysis, were not significantly different. The 3D strain analysis was extended in terms of principal and secondary strain (PS, SS). Global PS was very similar, global SS was significantly higher in athletes and displays a modified time course. The comparative analysis of strain-lines pattern suggests that the enhancement of LV function is achieved by a more synchronous recruitment of both left- and right-handed helical fibers.

**Conclusions:**

3D strain analysis allows a deeper physiological understanding of LV contraction in different types of athletes. Secondary strain, only available in 3D, identifies increase of performances due to physical activity; this appears to follow from the synergic activation of endocardial and epicardial fibers.

## Introduction

The modern approach for the Left Ventricle (LV) functional assessment has been recently supported by the development of noninvasive methods that allow investigating the relationship between myocardial contractile function and anatomical cardiac structure. Among these methods, 3D strain analysis presents the potential of evaluating the deformation of the 3D LV cavity in its entirety. Based on 3D strain information, very recently, the principal strain analysis (PSA), a method widely applied in engineering applications, was introduced in cardiac magnetic resonance [1,2] and lately in 3D echocardiography [3,4]. PSA provides a characterization of 3D strain properties, which includes longitudinal and circumferential strain values and torsional shear deformation, into a unitary picture. This is achieved by identification of the direction along which strain develops (so-called principal direction) and the entities of actual deformation along the principal direction and transversal to it. One of the field of application of the strain analysis is the athlete’s heart during all the phase of training. Regular physical exercise induces in fact myocardial morphological modifications recognized to be a consequence of the kind and intensity of the sports practiced [5,6]. Nevertheless, the understanding of the physiological counterpart of cardiac function associated to such structural modifications is still incomplete and a normal Ejection Fraction (EF) cannot be considered an exhaustive tool for the assessment of a normal myocardial function. This aspect can be particularly important in case young athletes, at the initial phase of their regular training and also in presence minor and very common cardiopathies as Bicuspid Aortic Valve (BAV) where the sport activity is normally allowed. An improved description of LV function can be useful particularly in those athletes, that do not manifest pathology in the early phases of their training.

In this study, we hypothesize that 3D strain and PSA may improve the physiological understanding of LV function and allows bridging with LV anatomical structure. Trained -but not elite- young athletic population is employed as a model to detect minor LV functional modifications. Athletes with BAV, with a normal LV function and trivial aortic valve insufficiency, are also included to reveal the presence of different functional changes paths.

## Methods

### Study population

Three homogeneous and age-matched groups of young (age 20.3 ± 5.4) male subjects are enrolled (see Table 1). One group of 15 athletes (TAV-Athletes), a second group of 20 BAV athletes (BAV-Athletes) with very mild aortic regurgitation, and a control group composed of 15 normal subjects (Controls). Both the BAV and TAV athletes were recruited from the athletic population, yearly followed up at Sports Medicine University Center in Florence (Italy). All athletes were selected among a large cohort in consideration of not being top elite athletes who were trained not intensively and since no more than 4 years before the observation. Additionally for the BAV group, the inclusion criteria were of being asymptomatic and to be regularly and yearly eligible for the agonistic sport activity and associated to a substantially normal aortic valve function. They were submitted to a similar exercise regime training (soccer, rugby, basketball and swimming), and presented a high quality of the echocardiographic acoustic window.

**Table 1:** **General and echocardiographic parameters of BAV**, **normals and TAV athletes**

		**Controls**	**TAV**-**Athletes**	**BAV**-**Athletes**
Age	[years]	19.1 ± 3.6	20.1 ± 3.5	21.4 ± 7.2
HR	[bpm]	70 ± 6.3	70.2 ± 11	66.1 ± 7.9
BMI	[Kg/m^2^ ]	21.7 ± 2.4	22.7 ± 2.5	22.6 ± 3.4
SBP	[mmHg]	114 ± 5.1	116.2 ± 6.5	115.8 ± 7.5
DBP	[mmHg]	74.7 ± 5.2^*x^	70 ± 4.1	69 ± 8
CMI	[g/m^2^]	103.3 ± 9.1	108.3 ± 17.1	113.7 ± 21.6
Ao Root	[mm]	29.6 ± 2.3^♣^	30.3 ± 2.2	32.9 ± 5.8
LA	[mm]	33.4 ± 2.4	33.1 ± 2.1	32.9 ± 3.1
IVS	[mm]	9.1 ± 0.6	9.3 ± 1	9.7 ± 1.3
PW	[mm]	9.1 ± 0.6	9.2 ± 0.9	9.4 ± 1.3
LVEDd	[mm]	50.3 ± 2.1	50.9 ± 3.7	51.2 ± 4.7
LVESd	[mm]	32 ± 2.6	32.1 ± 3.1	33 ± 3.6
EF	[%]	66.3 ± 4^+§^	66.3 ± 4	63.7 ± 2.6
E peak	[cm/s]	87 ± 16.5^‡#^	90.1 ± 18.3	75.9 ± 14.3
A peak	[cm/s]	53.5 ± 17.2	47.7 ± 11.9	46.1 ± 13.5
DT	[ms]	169.9 ± 19.9^†^	178.1 ± 28.3	201.2 ± 13.5
IVRT	[ms]	73.1 ± 8.1	76.6 ± 10.2	74 ± 13.5

Legend: *HR*: heart rate; *BMI*: body mass index; *SBP*: systolic blood pressure; *DBP*: diastolic blood pressure; Ao Root: aorta; *LA*: left atrium; *PW*: Posterior wall; *LVESd*: left ventricle end systolic diameter; *LVEDd*; left ventricle end diastolic diameter; *EF*: ejection fraction; *DT*: deceleration time; *IVRT*: isovolumetric relaxation time; Controls vs. TAV: *p = .014. Controls vs. BAV: ^x^p = .023, ^♣^p = .044, ^+^p = .026, ^‡^p = .040,^†^p = .006. TAV vs. BAV: ^§^p = .028, ^#^p = .018.

Athletes were regularly trained for at least 10 months a year. The training regimes for soccer and rugby players consisted of 12 ± 1.2 hours/week for 8.7 ± 2.5 years, for basketball players 14.5 ± 0.5 hours/week for 8.9 ± 2.3 years, for swimmers 15.5 ± 0.5 hours/week for 7.4 ± 0.5 years. The different kinds of sports practiced were all included as medium-high workload class if considered their dynamic and static sports components and following the task force classification dedicated to identify the responses of the cardiovascular system to the dynamic and static exercise [7]. The control group was engaged from a population of age-matched normal healthy sedentary subjects. All subjects were submitted to a clinical examination and to a conventional color Doppler echocardiographic exam performed following the AHA criteria [8]. Inclusion criteria were the absence of any symptom of dysfunction, metabolic diseases (as diabetes), hypertension or other cardiovascular disease including cardiomyopathies or pathological LV hypertrophy. An informed consent for participation to the study was obtained from all subjects or legal guardians.

### Standard 2D echocardiography

All echocardiographic exams were performed by two experienced certified cardiologists. From the standard long axis parasternal view, the standard M-mode echocardiographic systolic and diastolic LV chamber parameters were obtained [7], as well as the aortic root and the left atrium dimensions. From the apical view, the pulse wave Doppler data, at the mitral valve level, were measured to study the diastolic function. Basal measurements included inter ventricular septum (IVS) posterior wall (PW) thickness, left ventricle end diastolic diameter (LVEDd), left ventricle end-systolic diameter (LVESd), left atrium (LA) and aortic root (Ao) dimensions, pulse wave Doppler transmitral flow E-wave, A-wave (E/A ratio), deceleration time (DT), isovolumic relaxation time (IVRT). Any further additional parameter evaluating the diastolic function, like Tissue Doppler analysis (TDI) was considered only in case of evident alteration of the standard parameters and therefore the eventual calculation has been made just in case of exclusion of the subjects from the protocol study. The evaluation of LV Mass Index g/m^2^ (CMI) was obtained from Devereux procedure [9].

Considering the regular shape of the myocardial chamber and the complete absence of any symptom, the Ejection Fraction (EF) was estimated by LV diameters following the formula (LVEDd^3^–LVESd^3^)/LVEDd^3^. The degree of the valve insufficiency was described in first line by visual assessment determining the extension of the regurgitating jet on a 0 to 4+ scale by the color-flow mapping method from the four-chamber view [8]. Moreover, during the enrollment phase, a possible quantitative approach to identify and to exclude any eventual subjects with more severe aortic regurgitation was considered, using of vena contracta value, calculated from parasternal-long axis view [10].

### 2D Strain analysis

Images were obtained in the left lateral decubitus position, after a 15 minute rest using the echocardiograph Philips IE33 (Philips Healthcare, Andover, MA) equipped with a 2.5 MHz probe. The images were acquired in a cine loops triggered to the QRS complex, at a heart rate (HR) between 60 and 70 bps to avoid the effects of a large HR variability on the parameters. High quality images were acquired with excellent visualization of the endocardial and epicardial borders.

The 2D images had present a higher spatial and temporal resolution than 3D recording therefore strain measurement are, in principle, more accurate. On the other side, 2D analysis does not allow the complete perception of 3D deformation. The primary purpose of 2D strain analysis was here that of building a reference for validating the 3D analysis. To this purpose three apical B-mode sequences (2, 3, 4 chambers views) were recorded at optimal frame rate (>40 Hz) and stored in DICOM format for post-processing. Longitudinal strain (LS) was evaluated by the speckle/feature tracking technique at the sub-endocardial level. Analysis was performed with 2D-CPA software (TomTec Imaging System Gmbh, Unterschleissheim, Germany). Global values were then computed as averages from the 6 segments in each view. End-systolic values were defined for each projection at the instant when the volume, computed from that projection, reaches its minimum.

### 3D echocardiography and principal strain analysis

3D echocardiographic recording were performed at the highest possible frame rate (>20 frames per second) on a Philips IE33 ultrasound systems (Philips Healthcare, Andover, MA) taking care to ensure inclusion of the entire LV.

The LV motion, at the sub-endocardium level, is tracked by the 3D speckle/feature tracking technique with the 4D LV-Analysis software (TomTec Imaging System Gmbh, Unterschleissheim, Germany). The choice of focusing on the sub-endocardium, rather than on the entire myocardium, is dictated by the resolution of 3D echography that limits the possibility of effectively differentiating figures across the thickness as it solely consists of a few pixels. The same software automatically exported the tracked moving surface on all instants for further post-processing. The longitudinal strain (LS), circumferential strain (CS), and shear values are locally computed at every point on the exported surface. Strain and shear together completely describe the local deformation state with reference to the longitudinal and circumferential directions; however such a physical state can be equally evaluated referring to any arbitrary direction, among those, the principal direction is a special, useful one. The principal direction is defined such that the local deformation state can be described by two strain values and no shear: the principal strain (PS) is the deformation along the principal direction, and the secondary strain (SS) is transversal to it. In mathematical terms, PS and SS are the eigenvectors of the 2×2 matrix made of LS and CS on the diagonal and shear off-diagonal; technical details of the calculation process were previously published and are not repeated here [3,11].

3D global strain values were computed, for every strain component, as an average over the whole LV. 3D Strain was also measured as average over three levels (basal, median, apical), where the level division is made by dividing into three equal portions the base-apex length measured along the tissue at end diastole. End-systolic values were taken when the volume reaches its minimum value.

### Data variability assessment

The reliability of the 3D measurement was assessed by comparing of the 3D global longitudinal strain with the same values obtained by 2D analysis in 20 subjects. Inter-operator repeatability of the quantification process was assessed by evaluation of all strain component by two independent operators blind to each other’ results in 5 subjects, randomly selected.

### Statistical analysis

All continuous variables were reported as mean ± standard deviation. Paired Student t test was used for the comparison between 2D and 3D longitudinal strain and for the inter-operator evaluations. Unpaired Student t test was used for comparisons between two groups; analysis of variance (ANOVA) was used for comparison within the three groups. Statistical significance was assumed for p < 0.01; all values p < 0.05 are reported in the tables for completeness.

## Results

### Clinical and echocardiographic examination

The three homogeneous groups investigated are substantially similar for their general characteristics as shown in Table 1. Hemodynamic and echocardiographic parameters resulted within the normal range for all the subjects investigated, very similar among them considering their training regimen and age. Minor differences between the athletes groups (TAV and BAV) have been found with larger values of CMI associated with a larger dimension of the LV chamber in the latter; these difference were not statistically significant and known as peculiar for the BAV category. Controls presented slightly larger DBP values.

As expected, BAV-Athletes present some small differences with the other groups: Aortic root is wider, EF is just a little lower, although still in the normal range. Regarding the basic diastolic parameter, some slight differences have been found among the three groups: the E peak is a little lower in BAV athletes, despite within the normal range, while the only significant difference was detected in the DT measure that is more prolonged in BAV-Athletes with respect to Controls only (p < 0.01). All the diastolic parameters are maintained in the normal range. From the color Doppler analysis, the aortic valve insufficiency was found in BAV group only. As previously established in the inclusion criteria, the degree of the aortic valve insufficiency of BAV, estimated by color parameters, resulted compatible with a small or very mild insufficiency in all the subjects enrolled. No additional investigation by other color-Doppler parameters was therefore judged necessary.

### 3D strain

**Figure 1:**
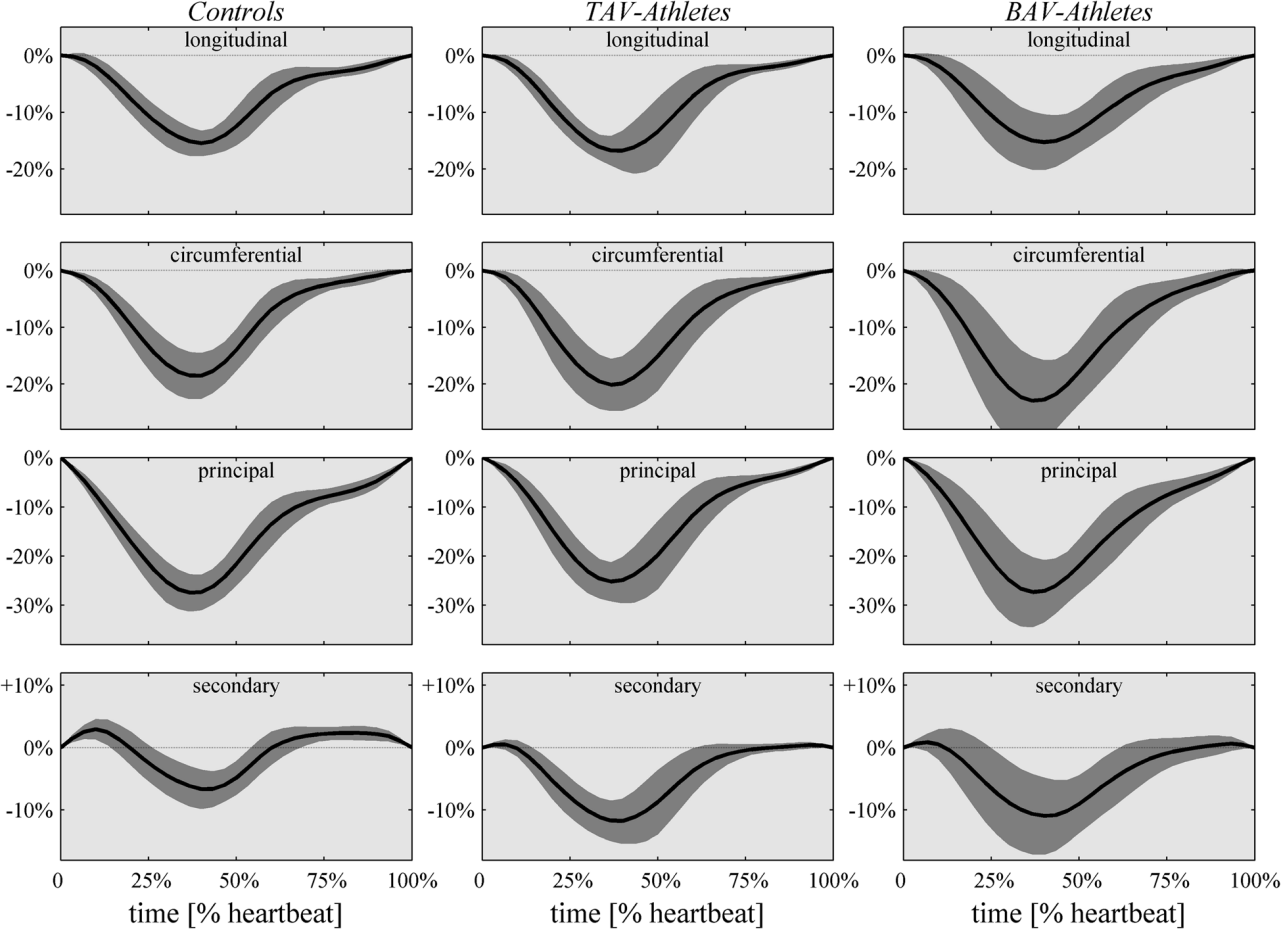
**Time course of the different global 3D strain components (from top to bottom, longitudinal, circumferential, principal and secondary) for the 3 groups reported as mean (black line) plus/minus standard deviation (shaded gray).** Longitudinal, circumferential and principal global strain curves are very similar in the three groups, with a noticeable small increase of circumferential strain in athletes. The secondary strain is markedly different, in particular the pre-stretch at the onset of contraction is reduced resulting in a higher end-systolic values.

End-systolic global 3D strain results are reported in Table 2. Global principal strain (GPS), longitudinal strain (GLS) and circumferential strain (GCS) are not significantly different among the three groups, despite a slightly larger GCS in BAV-Athletes. Global Secondary Strain (GSS), instead, is significantly higher in the athletes groups, both BAV and TAV, with respect of normal subjects (Controls: -6.6% ± 3.0, BAV-Athletes: -12.1% ± 6.3, TAV-Athletes: -12,8% ± 2.8; p = 0.005 and p < 0.001, respectively; p = 0.0015 in the ANOVA) and comparable between the two groups of athletes.The time course of the global strain components are shown in Figure 1 for the 3 groups. The curves for GLS, GCS and GPS are very similar in the three groups, with a small increase of circumferential strain in BAV-Athletes. The time curve of GSS shows that the higher end-systolic peak found in athletes is a consequence of the reduction of stretch transversally to the principal direction at the beginning of contraction.

**Table 2:** **Global end**-**systolic 3D strain values**

	**Controls**	**TAV**-**Athletes**	**BAV**-**Athletes**	**ANOVA**
GLS [%]	-16.2 ± 2.2^x^	-18.3 ± 2.5	-17.0 ± 4.6	p = 0.32
GCS [%]	-19.3 ± 3.8^*^	-21.8 ± 3.9	-25.5 ± 8.3	p = 0.02
GPS [%]	-28.4 ± 3.2	-27.2 ± 3.2	-30.0 ± 7.2	p = 0.34
GSS [%]	-6.6 ± 3.0^†‡^	-12.8 ± 2.8	-12.1 ± 6.3	p = 0.0015

Legend: *GLS* = end-systolic Global Longitudinal Strain; *GCS* = end-systolic Global Circumferential Strain; *GPS* = end-systolic Global Principal Strain; *GSS* = end-systolic Global Secondary Strain. Controls vs. TAV: ^x^p = 0.036; ^†^p < 0.001. Controls vs. BAV: ^*^p = .014; ^‡^p = .005.

**Figure 2:**
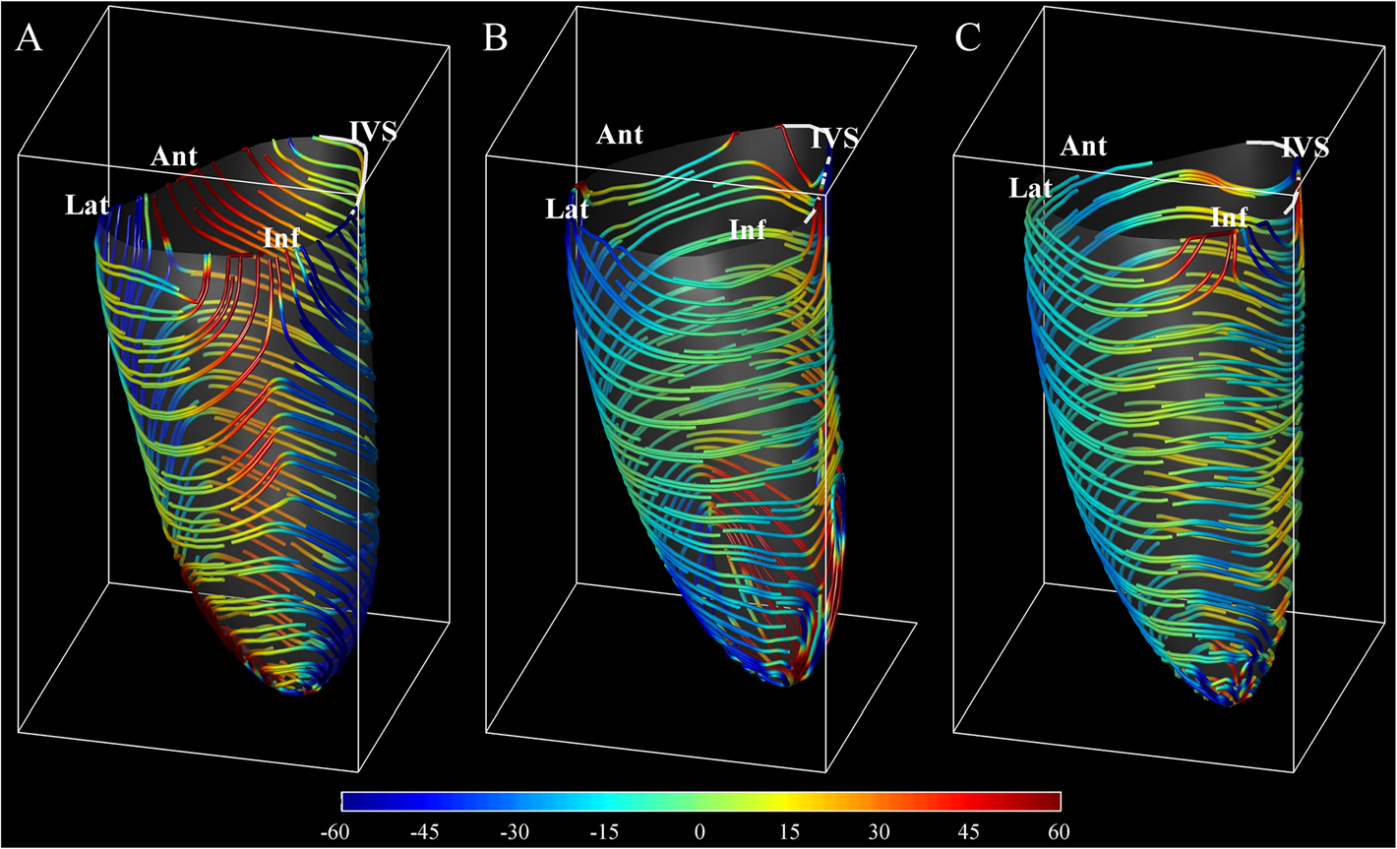
**Representation of principal strain direction at end**-**systole computed from the deformation at the sub**-**endocardial level averaged from the ( *A *) Controls group**, **( *B *) TAV**-**Athletes group**, **( *C *) BAV**-**Athletes group.** Strain lines are colored by their angle as by the colorbar on the bottom, to emphasize the positive angle of endocardial right-handed helical fibers (clockwise moving from base to apex) and negative angle for epicardial left-handed helix.

Strain values are also compared at the different levels in the LV. Following the results reported in Table 3, The TAV-Athletes have a higher than Controls CS at the median level and PS at the base; whereas BAV-Athletes present a higher than Control LS at the apical and median level. The increase of SS in both athletes groups is very significant at all levels with the exception of the basal level in BAV-Athletes.Results from PSA include the additional information about the direction along which principal strain develops and perpendicular to which secondary strain acts. The end-systolic principal strain-lines are reported in Figure 2 on average for the three-groups. Strain-lines in controls embrace around the septum with right-handed helical paths (analogous to those of endocardial fibers) descending from the anterior wall, and left-handed helical paths (similar to those of epicardial fibers) descending from the inferior wall. The behavior is specular around the lateral wall. In both groups of athletes strain-lines are more uniformly circumferential. The spatial distribution of strain (not shown here) is fairly uniform in all cases.

**Table 3:** **End**-**systolic strain parameters at different LV levels**

		**Controls**	**TAV**-**Athletes**	**BAV**-**Athletes**
LS [%]	Apex	-17.6 ± 3.0^x^	-23.4 ± 8.3	-20.6 ± 5.8
	Mid	-16.4 ± 2.1^♣^	-19.3 ± 3.3	-18.2 ± 5.1
	Base	-14.9 ± 2.9	-15.7 ± 2.7	-14.2 ± 4.5
CS [%]	Apex	-15.8 ± 3.7^§^	-19.7 ± 5.6	-26.1 ± 10.8
	Mid	-20.5 ± 4.5^‡^	-24.2 ± 4.5	-28.7 ± 10.6
	Base	-19.1 ± 3.8	-20.4 ± 4.5	-22.7 ± 6.5
PS [%]	Apex	-26.2 ± 4.1	-29.6 ± 8.1	-31.3 ± 9.5
	Mid	-28.9 ± 3.9	-29.3 ± 3.9	-32.7 ± 9.3
	Base	-28.8 ± 3.2^†^	-24.6 ± 3.3	-27.5 ± 5.9
SS [%]	Apex	-7.1 ± 2.8*^#^	-13.5 ± 4.2	-15.3 ± 8.5
	Mid	-7.9 ± 2.9*^§^	-14.2 ± 2.7	-14.3 ± 6.8
	Base	-5.2 ± 4.0*^+^	-11.4 ± 3.6	-9.4 ± 6

Legend: *LS* = end-systolic Longitudinal Strain; *CS* = end-systolic Circumferential Strain; *PS* = end-systolic Principal Strain; *SS* = end-systolic Secondary Strain. Controls vs. TAV: ^x^p = .023, ^♣^p = .011, ^†^p = .003, *p < .001. Controls vs. BAV: ^§^p = .002, ^‡^p = .010, ^#^p = .001, ^+^p = .03.

### Data variability assessment

Comparison between 2D and 3D GLS does not report significant differences (2D: -17.9 ± 2.3, 3D: -17.0 ± 4.6; p = 0.30). Inter-operator repeatability results for all 3D strain components, reported in Table 4, did not exhibit significant differences between the two independent operators.

**Table 4:** **Inter**-**operator reproducibility of 3D strain calculation in a group of 5 subjects measured by two independent operators**

	**Operator #1**	**Operator #2**	**p**
GLS [%]	-13.0 ± 1.9	-13.5 ± 2.3	0.87
GCS [%]	-18.5 ± 1.8	-19.9 ± 1.7	0.46
GPS [%]	-27.4 ± 1.0	-28.1 ± 2.4	0.58
GSS [%]	-3.6 ± 2.3	-5.3 ± 1.7	0.25

Legend: *GLS* = end-systolic Global Longitudinal Strain; *GCS* = end-systolic Global Circumferential Strain; *GPS* = end-systolic Global Principal Strain; *GSS* = end-systolic Global Secondary Strain.

## Discussion

The possibility of recognizing initial evidences of the myocardial remodeling due to a regular exercise can be difficult in young athletes. This aspect takes a particular relevance when the subjects investigated are affected by minor cardiopathies commonly compatible with a regular sport activity like BAV. Strain analysis, in its diverse components, can integrate morphological information with functional ones [11,12]. The results of the present study highlight that a 3D strain analysis has the ability to create a link between the mechanics of cardiac contraction and the underlying structure of cardiac fibers arrangement, especially in the initial phase of myocardial modifications. This improves the physiological understanding of myocardial contraction and its modifications induced, for example, by a regular sport training, despite non-intensive. The ability of differentiating functional changes due to training from pathological ones is of particularly relevance for young athletes, especially those with asymptomatic BAV [13], when a possible negative impact of regular physical exercise needs to be anticipated.

BAV young men usually undergo to cardiac evaluation for the eligibility to sport activity. Sports activity is normally allowed in presence of substantial normality of the myocardial performance as for the population selected for this study. However, this is a special kind of athletes where eligibility can be permitted exclusively under a periodical assessment of the valve and of the myocardial function. In this group, a deeper assessment of cardiac function can help in preventing adverse effects.

The results show an absence of substantial differences in the main deformation parameters, in the normal healthy group subjects and in both athletes groups, despite in presence of the slight differences in the LV dimensions. Form the data obtained the CS of the median LV segments are higher than of those found in the apex. This aspect can be partially in contrast with the literature where it has was reported that LV strain normally shows a peculiar gradient from base to apical segments. It must be considered that the current segmentation is based on the 3D endocardial surface and not on three projections; moreover, starting from the assumption that the apical segments can be considered as a portion of the medium-apical level, the numbers of the myocardial fibers are normally more represented in the medium level, especially in an athlete’s heart. A possible contribution due to a partial different distribution and any influence of the physical exercise on this aspect can be only hypothesized. This is in agreement with previous results where were functional changes at rest where limited and usually concurrent with morphological modifications. Normal strain levels were found in athletes with normal function and higher LV mass [14]; whereas higher values of strain [15] and torsion [16] were previously reported for athletes with larger LV volume and mass. On the contrary, a reduction of myocardial deformation with unmodified systolic function was suggested as a possible mechanism of reserve to respond to exercise [17]. The analysis of torsion represented a possibility to investigate additional aspects of LV contractile function; however, the current evaluation of torsion is prone to a variability due to the definition of two planes along which to evaluate the differential rotation. The usage of PSA in 3D echocardiography can circumvent this limitation including the effect of torsion (that is a component of shear) into the secondary components of strain [11]. PSA provides an evidence of the improvement of contractile activity in athletes where systolic function and standard strain values are unmodified and before appearances of morphological changes. In this respect, the SS parameter can be considered as particularly sensible in combining strain and shear along the longitudinal-circumferential reference in an appropriate physics-based manner, to investigate undetectable athlete’s heart modification when the systolic function is conserved. This is represented by the role of the secondary strain in the median-apical portion of the LV contributing to the enhancement of the heart’s performance. PSA suggests principal strain as a measure of the actual entity of contraction, thus its value tells whether systolic function is preserved or reduced. Secondary strain provides additional information about how such a contraction is achieved. This is a new aspect appears to be able of evidencing minor differences in LV contraction with unvaried systolic function, like that occurring with an initial physical activity but regularly practiced. The concurrent presence of normal principal strain and increased secondary strain together agrees with the previously documented increased torsional dynamics on the LV apex [18,19,17]. However, there are some contrasting evidences in this respects that acute maximal exercise can result to either an increase of LV systolic twisting mechanics or an impairment of systolic and diastolic twisting mechanics in endurance-trained individuals [20,21]. Secondary strain was previously shown to be a particularly sensible parameter to detect sub-clinical LV dysfunction in hypertensive subjects [11]. Here we show that is can also be a capable of detecting small early, possibly favorable, modifications of LV function in consequence of continuous, non-professional exercise.

The importance of the helical fiber orientation to cardiac performance has long been recognized [22]; the present PSA suggested a preliminary physiological picture on how different fibers participate to the normal contraction and to the increase of athletes’ performance.

The LV contraction in normal, non-athletic, subjects is characterized by a high principal strain and much lower secondary strain (<10%) [2,11]. The normal contraction is achieved with a different contribution of endocardial (right-handed helix) and epicardial (left-handed) fibers in the different regions. As shown in Figure 2*A*, right-handed deformation pattern (positive angle, red in the picture) from the anterior wall combines with left-handed pattern coming from the inferior wall to embrace the septal region; specularly, the lateral wall contraction is embraced between anterior left-handed and infero-lateral right-handed strain activity. The regional dominance of contraction along one specific helical direction testifies, the weakness of the activity along the counter-rotating helix. This, in turns, explains the positive value of secondary strain normally found at the beginning of contraction when the sudden contraction along one helical path provokes, for mass conservation, the stretching along counter-arranged fibers, temporarily inactive (shown in Figure 1).

Trained athletes, with no significant difference between TAV and BAV, do not display dominance of any helical contraction paths (Figure 2*B,C*) which means that myocardial activation develops over the entire thickness with a uniform and synchronous activation of both endocardial and epicardial fibers. Such a synergic activation reduces drastically the stretching along counter-arranged fibers, and therefore leads to increase of secondary strain. We can speculate that exercise trains the myocardial muscle across the entire thickness and, in spite of the normal function at rest, springs up the ability to reach higher performances detected in high-intensity exercise [23].

### Limitations

The study has been conducted in a relative small cohort of subjects, results should be considered as preliminary and the relevance of the new parameters, especially the secondary strain, will require further investigation in larger cohorts. This is particularly true for the BAV athletes population studied that needs a more long follow-up for evaluating the deformation parameters. However, the usage of limited population is common practice when exploring new approaches, and it was helpful in this respect that the groups were very homogeneous with little intra-group variability.

The low resolution and frame rate of 3D echocardiography may influence the quality of PSA results in terms of accuracy and reproducibility. We attempted to verify the reliability of 3D strain results by validating them with 2D results.

## Conclusions

The usage of the different components of 3D strain allows a deeper physiological understanding of LV contraction and its relationship with the LV structure. Secondary strain, only available in 3D, helps in detecting the increase of cardiac performances with physical activity. This appears to follow from the synergic activation of the counter-rotating helical fibers over entire myocardial thickness.

## Competing interest

The authors declare that they have no competing interests.

## Authors’ contributions

LS, GG, GP have planned the study, LS and GP have written the manuscript, ADL and LT have performed the post processing analysis of the images.ADL has contributed to the statistical analysis of the results. All authors read and approved the final manuscript.
